# Supplementary material for: Correlations between Molecular Alterations, Histopathological Characteristics, and Poor Prognosis in Esophageal Adenocarcinoma
Source: Cancers (Basel). 2023 Feb 23;15(5):1408. doi: 10.3390/cancers15051408 (PMC10000513; doi:10.3390/cancers15051408)
Supplement: Supplementary file 1 [file cancers-15-01408-s001.zip › cancers-2191970-supplementary.pdf]

Supplementary Table S1: *TP53* variant classification according to TP53 database.

<table>
<tr><th>TP53 variant</th><th>Domain function</th><th>Residue function</th><th>Effect</th><th>Damaging</th></tr>
<tr><td>Chr17: 7577104-A-AGGACAGGCACAAACAC</td><td>DNA binding</td><td>Buried</td><td>NA</td><td>NA</td></tr>
<tr><td>Chr17: 7578176-C-A | COSM118939</td><td>NA</td><td>NA</td><td>splice</td><td>NA</td></tr>
<tr><td>Chr17: 7578271-T-A | rs786201838</td><td>DNA binding</td><td>Buried</td><td>missense</td><td>Damaging</td></tr>
<tr><td>Chr17: 7577539-G-A | rs121912651</td><td>DNA binding</td><td>DNA binding</td><td>missense</td><td>Damaging</td></tr>
<tr><td>Chr17: 7577082-C-A | COSM10726</td><td>DNA binding</td><td>Partially exposed</td><td>nonsense</td><td>NA</td></tr>
<tr><td>Chr17: 7577548-C-T | rs28934575</td><td>DNA binding</td><td>Buried</td><td>missense</td><td>Damaging</td></tr>
<tr><td>Chr17: 7577114-C-T | rs863224451</td><td>DNA binding</td><td>DNA binding</td><td>missense</td><td>Damaging</td></tr>
<tr><td>Chr17: 7579314-TGCAAGTCACA-T</td><td>DNA binding</td><td>Buried</td><td>NA</td><td>NA</td></tr>
<tr><td>Chr17: 7577586-A-C | COSM10715</td><td>DNA binding</td><td>Buried</td><td>missense</td><td>Damaging</td></tr>
<tr><td>Chr17: 7578370-C-A | COSM131534</td><td>NA</td><td>NA</td><td>splice</td><td>NA</td></tr>
<tr><td>Chr17: 7577580-T-C | rs587780073</td><td>DNA binding</td><td>Buried</td><td>missense</td><td>Damaging</td></tr>
<tr><td>Chr17: 7578419-C-A | COSM10996</td><td>DNA binding</td><td>Partially exposed</td><td>nonsense</td><td>NA</td></tr>
<tr><td>Chr17: 7578212-G-A | rs397516436</td><td>DNA binding</td><td>Buried</td><td>nonsense</td><td>NA</td></tr>
<tr><td>Chr17: 7579346-AAGAAGCCC-A</td><td>DNA binding</td><td>NA</td><td>NA</td><td>NA</td></tr>
<tr><td>Chr17: 7578509-A-G | rs1057519978</td><td>DNA binding</td><td>-glutathionylation sit</td><td>missense</td><td>Damaging</td></tr>
<tr><td>Chr17: 7578272-G-A | rs876658468</td><td>DNA binding</td><td>Buried</td><td>missense</td><td>Damaging</td></tr>
<tr><td>Chr17: 7577094-G-A | rs28934574</td><td>DNA binding</td><td>Buried</td><td>missense</td><td>Damaging</td></tr>
<tr><td>Chr17: 7578380-C-CT</td><td>DNA binding</td><td>Exposed</td><td>missense</td><td>Damaging</td></tr>
<tr><td>Chr17: 7579326-A-ACTTGGCTG</td><td>DNA binding</td><td>DNA binding</td><td>NA</td><td>NA</td></tr>
<tr><td>Chr17: 7579506-C-CTGGACCTGGGTCTTCAG</td><td>SH3-like/Pro-rich</td><td>NA</td><td>NA</td><td>NA</td></tr>
<tr><td>Chr17: 7577094-G-A | rs28934574</td><td>DNA binding</td><td>Buried</td><td>missense</td><td>Damaging</td></tr>
<tr><td>Chr17: 7577539-G-A | rs121912651</td><td>DNA binding</td><td>DNA binding</td><td>missense</td><td>Damaging</td></tr>
<tr><td>Chr17: 7578463-G-C</td><td>DNA binding</td><td>Partially exposed</td><td>missense</td><td>Tolerated</td></tr>
<tr><td>Chr17: 7577539-G-A | rs121912651</td><td>DNA binding</td><td>DNA binding</td><td>missense</td><td>Damaging</td></tr>
<tr><td>Chr17: 7578196-A-C | COSM44198</td><td>DNA binding</td><td>Buried</td><td>missense</td><td>Damaging</td></tr>
<tr><td>Chr17: 7577539-G-A | rs121912651</td><td>DNA binding</td><td>DNA binding</td><td>missense</td><td>Damaging</td></tr>
<tr><td>Chr17: 7577094-G-A | rs28934574</td><td>DNA binding</td><td>Buried</td><td>missense</td><td>Damaging</td></tr>
<tr><td>Chr17: 7578525-G-C | rs1057519976</td><td>DNA binding</td><td>Buried</td><td>missense</td><td>Damaging</td></tr>
<tr><td>Chr17: 7577117-A-G | COSM131453</td><td>DNA binding</td><td>Buried</td><td>missense</td><td>Damaging</td></tr>
<tr><td>Chr17: 7577550-C-T | rs1057517983</td><td>DNA binding</td><td>Exposed</td><td>missense</td><td>Damaging</td></tr>
<tr><td>Chr17: 7578271-T-C | rs786201838</td><td>DNA binding</td><td>Buried</td><td>missense</td><td>Damaging</td></tr>
<tr><td>Chr17: 7578269-G-A | rs587780071</td><td>DNA binding</td><td>Buried</td><td>missense</td><td>Damaging</td></tr>
<tr><td>Chr17: 7577105-G-A | rs876659802</td><td>DNA binding</td><td>Buried</td><td>missense</td><td>Damaging</td></tr>
<tr><td>Chr17: 7579722-C-T</td><td>NA</td><td>NA</td><td>splice</td><td>NA</td></tr>
<tr><td>Chr17: 7578542-G-C | rs863224683</td><td>DNA binding</td><td>Partially exposed</td><td>missense</td><td>Damaging</td></tr>
<tr><td>Chr17: 7579312-C-A | rs55863639</td><td>DNA binding</td><td>Buried</td><td>splice</td><td>Tolerated</td></tr>
<tr><td>Chr17: 7577106-G-A | COSM10814</td><td>DNA binding</td><td>Buried</td><td>missense</td><td>Damaging</td></tr>
<tr><td>Chr17: 7577120-C-T | rs28934576</td><td>DNA binding</td><td>DNA binding</td><td>missense</td><td>Damaging</td></tr>
<tr><td>Chr17: 7579374-C-A</td><td>DNA binding</td><td>Buried</td><td>missense</td><td>Damaging</td></tr>
<tr><td>Chr17: 7577538-C-T | rs11540652</td><td>DNA binding</td><td>DNA binding</td><td>missense</td><td>Damaging</td></tr>
<tr><td>Chr17: 7579368-A-C | COSM220765</td><td>DNA binding</td><td>NA</td><td>missense</td><td>Damaging</td></tr>
<tr><td>Chr17: 7577547-C-T | rs121912656</td><td>DNA binding</td><td>Buried</td><td>missense</td><td>Damaging</td></tr>
<tr><td>Chr17: 7578524-G-C | COSM11166</td><td>DNA binding</td><td>Buried</td><td>missense</td><td>Damaging</td></tr>
<tr><td>Chr17: 7578475-G-A | rs587782705</td><td>DNA binding</td><td>Partially exposed</td><td>missense</td><td>Damaging</td></tr>
<tr><td>Chr17: 7578212-G-A | rs397516436</td><td>DNA binding</td><td>Buried</td><td>nonsense</td><td>NA</td></tr>
<tr><td>Chr17: 7577120-C-T | rs28934576</td><td>DNA binding</td><td>DNA binding</td><td>missense</td><td>Damaging</td></tr>
<tr><td>Chr17: 7578197-C-CCACCA</td><td>DNA binding</td><td>Buried</td><td>FS</td><td>NA</td></tr>
<tr><td>Chr17: 7578265-A-G | rs760043106</td><td>DNA binding</td><td>Buried</td><td>missense</td><td>Damaging</td></tr>
<tr><td>Chr17: 7578555-C-T | rs868137297</td><td>NA</td><td>NA</td><td>splice</td><td>NA</td></tr>
<tr><td>Chr17: 7574003-G-A | rs730882029</td><td>Tetramerisation/NES</td><td>NA</td><td>nonsense</td><td>NA</td></tr>
<tr><td>Chr17: 7577156-C-T | COSM127199</td><td>NA</td><td>NA</td><td>splice</td><td>NA</td></tr>
<tr><td>Chr17: 7578406-C-T | rs28934578</td><td>DNA binding</td><td>Buried</td><td>missense</td><td>Damaging</td></tr>
<tr><td>Chr17: 7579329-T-C | rs121912658</td><td>DNA binding</td><td>ylation/Ubiquitination</td><td>missense</td><td>Damaging</td></tr>
<tr><td>Chr17: 7578403-C-A | COSM10645</td><td>DNA binding</td><td>Zn binding</td><td>missense</td><td>Damaging</td></tr>
<tr><td>Chr17: 7577106-G-T | COSM10814</td><td>DNA binding</td><td>Buried</td><td>missense</td><td>Damaging</td></tr>
<tr><td>Chr17: 7577081-T-A | rs1057519985</td><td>DNA binding</td><td>Partially exposed</td><td>missense</td><td>Damaging</td></tr>
<tr><td>Chr17: 7577538-C-T | rs11540652</td><td>DNA binding</td><td>DNA binding</td><td>missense</td><td>Damaging</td></tr>
<tr><td>Chr17: 7577539-G-A | rs121912651</td><td>DNA binding</td><td>DNA binding</td><td>missense</td><td>Damaging</td></tr>
<tr><td>Chr17: 7578475-G-A | rs587782705</td><td>DNA binding</td><td>Partially exposed</td><td>missense</td><td>Damaging</td></tr>
<tr><td>Chr17: 7577121-G-A | rs121913343</td><td>DNA binding</td><td>DNA binding</td><td>missense</td><td>Damaging</td></tr>
<tr><td>Ch17: 7578460-A-C | COSM1480073</td><td>DNA binding</td><td>Buried</td><td>missense</td><td>Damaging</td></tr>
<tr><td>Chr17: 7578550-G-T | COSM1637542</td><td>DNA binding</td><td>Buried</td><td>missense</td><td>Damaging</td></tr>
<tr><td>Chr17: 7577538-C-T | rs11540652</td><td>DNA binding</td><td>DNA binding</td><td>missense</td><td>Damaging</td></tr>
<tr><td>Chr17: 7578406-C-T | rs28934578</td><td>DNA binding</td><td>Buried</td><td>missense</td><td>Damaging</td></tr>
<tr><td>Chr17: 7577121-G-A | rs121913343</td><td>DNA binding</td><td>DNA binding</td><td>missense</td><td>Damaging</td></tr>
<tr><td>Chr17: 7577574-T-C | COSM10731</td><td>DNA binding</td><td>Buried</td><td>missense</td><td>Damaging</td></tr>
<tr><td>Chr17: 7578452-TG-T | COSM44130</td><td>DNA binding</td><td>Buried</td><td>FS</td><td>NA</td></tr>
<tr><td>Chr17: 7578406-C-T | rs28934578</td><td>DNA binding</td><td>Buried</td><td>missense</td><td>Damaging</td></tr>
<tr><td>Chr17: 7577538-C-T | rs11540652</td><td>DNA binding</td><td>DNA binding</td><td>missense</td><td>Damaging</td></tr>
<tr><td>Chr17: 7578431-G-A | COSM11333</td><td>DNA binding</td><td>Exposed</td><td>nonsense</td><td>NA</td></tr>
<tr><td>Chr17: 7577538-C-T | rs11540652</td><td>DNA binding</td><td>DNA binding</td><td>missense</td><td>Damaging</td></tr>
<tr><td>Chr17: 7578406-C-T | rs28934578</td><td>DNA binding</td><td>Buried</td><td>missense</td><td>Damaging</td></tr>
<tr><td>Chr17: 7578256-TC-T | COSM118010</td><td>DNA binding</td><td>Partially exposed</td><td>FS</td><td>NA</td></tr>
<tr><td>Chr17: 7578291-T-C | COSM1679503</td><td>NA</td><td>NA</td><td>splice</td><td>NA</td></tr>
<tr><td>Chr17: 7577550-C-T | rs1057517983</td><td>DNA binding</td><td>Exposed</td><td>missense</td><td>Damaging</td></tr>
<tr><td>Chr17: 7578394-T-C | rs1057519991</td><td>DNA binding</td><td>Zn binding</td><td>missense</td><td>Damaging</td></tr>
<tr><td>Chr17: 7579311-C-A | COSM127204</td><td>NA</td><td>NA</td><td>splice</td><td>NA</td></tr>
<tr><td>Chr17: 7578406-C-T | rs28934578</td><td>DNA binding</td><td>Buried</td><td>missense</td><td>Damaging</td></tr>
<tr><td>Chr17: 7577094-G-A | rs28934574</td><td>DNA binding</td><td>Buried</td><td>missense</td><td>Damaging</td></tr>
<tr><td>Chr17: 7577538-C-T | rs11540652</td><td>DNA binding</td><td>DNA binding</td><td>missense</td><td>Damaging</td></tr>
<tr><td>Chr17: 7577141-C-A | rs193920774</td><td>DNA binding</td><td>Buried</td><td>missense</td><td>Damaging</td></tr>
<tr><td>Chr17: 7578403-C-A | COSM10645</td><td>DNA binding</td><td>Zn binding</td><td>missense</td><td>Damaging</td></tr>
<tr><td>Chr17: 7577097-C-T | rs764146326</td><td>DNA binding</td><td>Buried</td><td>missense</td><td>Damaging</td></tr>
<tr><td>Chr17: 7578437-G-A</td><td>DNA binding</td><td>Partially exposed</td><td>nonsense</td><td>NA</td></tr>
<tr><td>Chr17: 7578406-C-T | rs28934578</td><td>DNA binding</td><td>Buried</td><td>missense</td><td>Damaging</td></tr>
<tr><td>Chr17: 7577121-G-A | rs121913343</td><td>DNA binding</td><td>DNA binding</td><td>missense</td><td>Damaging</td></tr>
<tr><td>Chr17: 7578534-C-A | rs866775781</td><td>DNA binding</td><td>Ubiquitination site</td><td>missense</td><td>Damaging</td></tr>
<tr><td>Chr17: 7578280-G-A | COSM100027</td><td>DNA binding</td><td>Partially exposed</td><td>missense</td><td>Damaging</td></tr>
<tr><td>Chr17: 7578479-G-C | COSM10905</td><td>DNA binding</td><td>Buried</td><td>missense</td><td>Damaging</td></tr>
<tr><td>Chr17: 7577599-CAG-C | COSM46164</td><td>DNA binding</td><td>Exposed</td><td>FS</td><td>NA</td></tr>
<tr><td>Chr17: 7577511-A-AGT</td><td>DNA binding</td><td>Buried</td><td>NA</td><td>NA</td></tr>
<tr><td>Chr17: 7577517-A-T | rs876659675</td><td>DNA binding</td><td>ADP-ribosylation site</td><td>missense</td><td>Damaging</td></tr>
<tr><td>Chr17: 7578263-G-A | rs397516435</td><td>DNA binding</td><td>Buried</td><td>nonsense</td><td>NA</td></tr>
<tr><td>Chr17: 7577539-G-A | rs121912651</td><td>DNA binding</td><td>DNA binding</td><td>missense</td><td>Damaging</td></tr>
<tr><td>Chr17: 7578212-GA-A | rs864309495</td><td>DNA binding</td><td>Buried</td><td>FS</td><td>NA</td></tr>
<tr><td>Chr17: 7578433-G-C | COSM11508</td><td>DNA binding</td><td>Exposed</td><td>nonsense</td><td>NA</td></tr>
<tr><td>Chr17: 7577539-G-A | rs121912651</td><td>DNA binding</td><td>DNA binding</td><td>missense</td><td>Damaging</td></tr>
<tr><td>Chr17: 7579532-T-TG</td><td>Transactivation TAD2</td><td>NA</td><td>NA</td><td>NA</td></tr>
<tr><td>Chr17: 7578202-A-C | COSM119678</td><td>DNA binding</td><td>Buried</td><td>missense</td><td>Damaging</td></tr>
<tr><td>Chr17: 7579591-C-T | COSM1610880</td><td>NA</td><td>NA</td><td>splice</td><td>NA</td></tr>
<tr><td>Chr17: 7578211-C-T | rs587778720</td><td>DNA binding</td><td>Buried</td><td>missense</td><td>Damaging</td></tr>
<tr><td>Chr17: 7578508-C-T | rs587781288</td><td>DNA binding</td><td>-glutathionylation sit</td><td>missense</td><td>Damaging</td></tr>
<tr><td>Chr17: 7578406-C-T | rs28934578</td><td>DNA binding</td><td>Buried</td><td>missense</td><td>Damaging</td></tr>
<tr><td>Chr17: 7579470-CG-C | COSM1268331</td><td>SH3-like/Pro-rich</td><td>NA</td><td>FS</td><td>NA</td></tr>
<tr><td>Chr17: 7577538-C-T | rs11540652</td><td>DNA binding</td><td>DNA binding</td><td>missense</td><td>Damaging</td></tr>
<tr><td>Chr17: 7579532-T-TG</td><td>Transactivation TAD2</td><td>NA</td><td>NA</td><td>NA</td></tr>
<tr><td>Chr17: 7579349-A-C | COSM10717</td><td>DNA binding</td><td>Buried</td><td>missense</td><td>Damaging</td></tr>
<tr><td>Chr17: 7578217-G-A | COSM1386676</td><td>DNA binding</td><td>Phosphorylation site</td><td>missense</td><td>Damaging</td></tr>
<tr><td>Chr17: 7578526-C-A | COSM10647</td><td>DNA binding</td><td>Buried</td><td>missense</td><td>Damaging</td></tr>
<tr><td>Chr17: 7578290-C-G | COSM127200</td><td>NA</td><td>NA</td><td>splice</td><td>NA</td></tr>
</table>

Supplementary Table S2: Degree of staining for HNF1alpha and % of variant alleles in tumors.
IHC: 1+ weak positive staining, 2+ strong positive staining. Results in each column are reported as % of positive cells. Sample EAC_15 was used as positive. Control for staining, as it did not carry any *HNF1alpha* variants.

EAC ID	HNF1alpha variations	HNF1A reads	gnomAD or COSMIC database	Other genes mutated	HNF1A-IHC 1+ %	HNF1A-IHC 2+ %	pT	pN
150	p.Gly292ArgfsTer25	12/122 (9.836%)	rs751449138	none	50	50	3	1
191	p.Pro337Leu	896/1163 (77,04%)	rs56031130	ATM, TP53,	30	0	3	3
192	p.Ala161Thr	778/1703 (46%)	rs201095611	PIK3CA	2	0	2	1
203	p.Ala161Thr	587/1344 (44%)	rs201095611	TP53, PIK3CA	15	0	3	2
204	p.Pro141Ser / p.Pro379LeufsTer5	57/374 (15%) / 114/401 (28%)	rs150513055 / COSM2175480	SMAD4, IDH2, RET, CTNNB1, FLT3, MET, MSH6, PIK3CA	80	0	2	1
226	p.Arg168His	957/1208 (79%)	rs377110124	ATM, ERBB2, CTNNB1	2	0	3	3
233	p.His505Asn	518/767 (68%)	rs577078110	EGFR	10	0	4	3
15	wt				50	0	2	1

Supplementary Table S3: Correlation analyses for the different gene mutations found in 164 EAC cases.

Gene 1	Gene 2	Pearson Correlation Coefficient	p-value
TP53	*ATM*	-0.147	0.047
TP53	*HNF1A*	-0.166	0.031
TP53	*MET*	-0.195	0.011
MSH6	*PIK3CA*	0.152	0.048
ERBB2	*KRAS*	0.153	0.047
CTNNB1	*IDH2*	0.155	0.044
MET	*IDH2*	0.155	0.044
SMARCA4	*ERBB2*	0.155	0.044
MAP2K1	*PIK3CA*	0.158	0.040
TP53	*CDKN2A*	0.162	0.035
CHEK2	*ERBB2*	0.163	0.034
CHEK2	*SMARCA4*	0.163	0.034
HNF1A	*RET*	0.163	0.034
RET	*FLT3*	0.163	0.034
RET	*HNF1A*	0.163	0.034
ALK	*CDKN2A*	0.174	0.024
CTNNB1	*SMAD4*	0.174	0.023
MET	*SMAD4*	0.174	0.023
STK11	*ALK*	0.176	0.022
STK11	*IDH2*	0.176	0.022
CTNNB1	*RET*	0.180	0.019
MET	*RET*	0.180	0.039
MSH6	*ERBB2*	0.185	0.032
MSH6	*RET*	0.191	0.013
CHEK2	*EGFR*	0.197	0.010
HNF1A	*EGFR*	0.197	0.010
EGFR	*CHEK2*	0.197	0.010
SMAD4	*IDH2*	0.201	*0.009*

RET	*IDH2*	0.203	*0.008*
SMARCA4	*STK11*	0.212	*0.006*
CDK6	*APC*	0.221	*0.004*
APC	*CDK6*	0.221	*0.004*
PIK3CA	*SMARCA4*	0.222	*0.004*
SMARCA4	*PIK3CA*	0.222	0.040
PIK3CA	*PTEN*	0.224	*0.003*
PTEN	*KRAS*	0.233	*0.002*
MET	*MSH6*	0.235	*0.002*
MSH6	*CTNNB1*	0.235	*0.002*
ARID2	*IDH2*	0.241	*0.002*
STK11	*ARID2*	0.241	*0.002*
HNF1A	*PIK3CA*	0.248	*0.000*
PIK3CA	*MSH6*	0.249	*0.001*
SMARCA4	*MSH6*	0.249	*0.001*
MAP2K1	*FLT3*	0.252	*0.001*
SMARCA4	*KRAS*	0.254	*0.000*
CTNNB1	*PTEN*	0.258	*0.001*
CTNNB1	*KRAS*	0.281	*0.000*
HNF1A	*CTNNB1*	0.281	*0.000*
CTNNB1	*SMARCA4*	0.305	*0.000*
MET	*CTNNB1*	0.309	*0.000*
PIK3CA	*KRAS*	0.353	*0.000*
PIK3CA	*CTNNB1*	0.390	*0.000*
ARID2	*CHEK2*	0.422	*0.000*

Supplementary Table S4: Cancer-specific survival and recurrence for the different types of *TP53* mutations. Crosstabulation analysis using Chi-square test with Pearson's correction, degrees of freedom=3).

a) Cancer-specific survival (CSS) in the EAC cases.

			**CSS**		
			0	1	Total
***TP53* mutations**	wild-type	Count (%)	42 (40.4)	17 (28.3)	59
	Missense	Count (%)	38 (36.5)	35 (58.3)	73
	LOF	Count (%)	21 (20.2)	8 (13.3)	29
	missense+LOF	Count (%)	3 (2.9%)	0	3
	Total		104	60	164
Pearson's Chi-square=8.340		***P*=0.039**			

b) Recurrence in the EAC cases

			**Recurrence**		
			0	1	Total
***TP53* mutations**	wild-type	Count (%)	37 (39.8)	22 (31)	59
	Missense	Count (%)	33 (35.5)	40 (56.3)	73
	LOF	Count (%)	20 (21.5)	9 (12.7)	29
	missense+LOF	Count (%)	3 (3.2)	0	3
	Total		93	71	164
Pearson's Chi-square=8.866		***P*=0.031**			

c) correlation between EACGSE risk classification (Lower and Higher Risks) and *TP53* mutations (df=1).

			EACGSE risk		
***TP53* mutations**			Lower risk	Higher risk	Total
	No	Count (%)	28 (47.5)	31 (52.5)	59
	Yes	Count (%)	31 (52.5.5)	74 (70.5)	105
	Total		59	105	164
Pearson's Chi-square=5.275		***P*=0.022**			

Supplementary Table S5. Cancer-specific survival, *TP53* mutations and EACGSE classification.

a) CSS and EACGSE risk (Kaplan Meier analysis).

	**EACGSE risk**	**Total N**	**N events**	**Censored**	
				N	%
wild-type	Lower risk	35	9	26	74.3
	Higher risk	38	17	21	55.3
	Overall	73	26	47	64.4
Missense	Lower risk	30	8	22	73.3
	Higher risk	62	32	30	48.4
	Overall	92	40	52	56.5
LOF	Lower risk	11	3	8	72.7
	Higher risk	21	7	14	66.7
	Overall	32	10	22	68.8
missense + LOF	Lower risk	2	0	2	100
	Higher risk	2	1	2	66.7
	Overall	5	1	4	80.0
	**Total**	202	77	125	61.9

		**Chi-Square**	**DF**	***P***
wild-type	Log Rank (Mantel-Cox)	2.062	1	0.151
Missense	Log Rank (Mantel-Cox)	11.880	1	**0.001**
LOF	Log Rank (Mantel-Cox)	0.488	1	0.485
Both missense and LOF	Log Rank (Mantel-Cox)	0.500	1	0.480

b) CSS, *TP53* mutations and EACGSE morphological classification (Kaplan Meier analysis).

	**EACGSE classification**	**Total N**	**N events**	**Censored**	
				N	%
wild-type	GL WD	28	7	21	75
	GL PD	22	9	13	59.1
	DDC	4	1	3	75
	DAC	4	3	1	25
	IMC	5	1	4	80
	MMC	3	1	2	66.7
	MIX	7	4	3	42.9
	Overall	73	26	47	64.4
Missense	GL WD	28	8	20	71.4
	GL PD	46	23	23	50
	DAC	3	1	2	66.7
	IMC	6	3	3	50
	MMC	2	0	2	100
	MIX	7	5	2	28.6
	Overall	92	40	52	56.5
LOF	GL WD	10	3	7	70
	GL PD	18	7	11	61.1
	IMC	1	0	1	100
	MMC	1	0	1	100

	MIX	2	0	2	100
	Overall	32	10	22	68.8
Both missense and LOF	GL WD	2	0	2	100
	GL PD	3	1	2	66.7
	Overall	5	1	4	80.0
	**Total**	202	77	125	61.9

		**Chi-Square**	**DF**	***P***
Wild-type	Log Rank (Mantel-Cox)	4.286	6	0.638
Missense	Log Rank (Mantel-Cox)	23.505	5	**0.000 (10^{-3})**
LOF	Log Rank (Mantel-Cox)	1.165	4	0.889
Both missense and LOF	Log Rank (Mantel-Cox)	0.500	1	0.480

c) Correlation with age

Case Summaries

Age

TP53	N	Median	Mean	Std. Deviation	Minimum	Maximum
Missense	73	71,00	67,67	13,190	23	85
LOF	29	76,00	73,07	8,892	50	85
Missense + LOF	3	84,00	80,67	5,774	74	84
Total	105	73,00	69,53	12,323	23	85

Kruskal-Wallis test, *P*=0.029.

Supplementary Table S6: Correlations between *TP53* mutations and p53 immunostaining.

		**Total N**	**N events**	**Censored**	
				N	%
wild-type	Lower risk	35	9	26	74.3
	Higher risk	38	17	21	55.3
	Overall	73	26	47	64.4
Missense	Lower risk	30	8	22	73.3
	Higher risk	62	32	30	48.4
	Overall	92	40	52	56.5
LOF	Lower risk	11	3	8	72.7
	Higher risk	21	7	14	66.7
	Overall	32	10	22	68.8
missense + LOF	Lower risk	2	0	2	100
	Higher risk	2	1	2	66.7
	Overall	5	1	4	80.0
	**Total**	202	77	125	61.9

Supplementary Table S7: Spearman's correlation coefficient for *TP53* mutation types and p53 immunostaining patterns (IHC).*TP53* mutations: 0=wild-type, 1=missense, 2=LOF, p53 immunostaining: 0=normal expression, 1=overexpression, 2= null expression).

			TP53 mutation (0=wild-type; 1=missense; 2=LOF)	IHC for p53
Spearman's rho	*TP53* mutations (0=wild-type 1=missense 2=LOF)	Correlation Coefficient	1.000	**0.782****
		Sig. (2-tailed)		0.000
		N	164	145
	IHC for p53	Correlation Coefficient	**0.782**	1.000
		Sig. (2-tailed)		0.000
		N	145	145

** Correlation is significant at the 0.01 level (2-tailed).

Supplementary Table S8: Correlation between *TP53* LOF mutations and loss of SMAD4 immunostaining. (Mann-Whitney's test; grouping variable: type of TP53 mutations)

	***TP53* mutations**	**N**	**Mean Rank**	**Sum of Rank**
**SMAD4 loss**	wild-type	70	45.57	3190.00
	LOF	30	62.00	1860.00
	Total	100		

	**SMAD4 loss**
Mann-Whitney U	705.000
Wilcoxon W	3190.000
Z	-2.661
Asymp. Sig. (2-tailed)	0.008

Supplementary Table S9: Cox regression analyses for CSS and clinical, genetic, pathological variables.

a) Univariate Cox regression analysis

	P	HR	95% CI
TP53 status	0.733	1.066	0.737-1.543
SMAD4 loss (cut-off 35)	0.942	1.022	0.571-1.830
Sex	0.995	0.998	0.505-1.971
Age	*0.028*	1.024	1.003-1.046
Stage	*0.001*	1.950	1.307-2.912
Lymph node ratio	*0.000*	11.28	4.626-27.513
EACSGE Risk	*0.003*	2.512	1.359-4.645

b) Multivariate Cox regression analysis (only significant variable are shown)

	P	HR	95% CI
Age	0.005	1.030	1.009-1.052
Lymph node ratio	0.000	8.911	3.299-24.064
EACSGE Risk	0.023	2.202	1.114-4.355

Supplementary Table S10: Gene fusions detected in EAC samples. Chromosomal position (in bp) of break points reported according to hg19. The gene-fusions confirmed in Sanger sequencing are indicated in bold. In two samples we detected two different gene fusions (see text for details).

EAC ID	Gene fusion identified	Chr_1	Break_1	Chr_2	Break_2	EACSGE classification	EACSGE subgroup	*TP53* status	Sanger confirmed
**EAC_197**	CYP2C19_CYP2C18	10	96541752	10	96493052	GL PD	High grade	p.Asp208Glu	**YES**
	GIPC1_DNAJB1	19	14602467	19	14627858				**YES**
**EAC_198**	IQCE_DGKB	7	2629740	7	14385016	GL WD	Low grade	p.Ala138Val	**YES**
**EAC_199**	PI4KA_MAPK1	22	21096516	22	22153417	GL WD	Low grade	wild-type	**YES**
	CYP2C19_CYP2C18	10	96541752	10	96493052				**YES**
**EAC_209**	FRYL_PI4KA	4	48686680	22	21104293	GL PD	High grade	p.Arg273Leu - pGln192Ter	**YES**
EAC_210	*AC073283.4-EPCAM*	*2*	*47572040*	*2*	*47606092*	*GL PD*	*High grade*	*wild-type*	*NO*
	LOC101927043-EPCAM	*2*	*47572039*	*2*	*47606091*				*NO*
EAC_298	*TPRG1_LPP*	*3*	*188850486*	*3*	*188242453*	*DIFF ANAPL*		*p.Cys135Trp*	*NO*
